# American Dental Association

**Published:** 1887-12

**Authors:** 


### AMERICAN DENTAL ASSOCIATION.

### TWENTY-SEVENTH ANNUAL MEETING.

Reported for the Independent Practitioner

Continued from Page 587.

Section VI, "Physiology and Etiology," was called. Dr. H. A. Smith, the Chairman, reported that the Secretary of the Section was absent, and consequently the formal report which he should have presented was lacking. The Section desired to present two papers for the consideration of the Association, one by Dr. Ottofy, of Chicago, and the other by Dr. Peirce, of Philadelphia. The Section also presented a report reviewing the action of the Association in the matter of the two hundred-dollar prize offered for the best paper upon the Etiology of Dental Caries, at the meeting in 1882, and which was declared awarded to Dr. W. D. Miller, of Berlin, at the meeting of 1883, but which award was, by a small minority, at the close of the session, when nearly all the members had left, reconsidered. The report had been very carefully considered in the Section, and the members were unanimously of the opinion that the honor of the Association was involved, and it recommended the passage of a resolution reaffirming the award, and directing the Treasurer to pay the sum of two hundred dollars to Dr. Miller in accordance with the original action of the Association. The resolution was passed unanimously.

### THURSDAY MORNING SESSION.

The regular order of the day was the selection of a place of meeting for 1888, and the election of officers. Drs. Reed, of Chicago, and Lucky, of Paterson, N. J., were appointed tellers.

Dr. Winder said that it had been suggested that the Association meet with the Southern Association at some point in the Southern States. There are but two or three places in the South which present the attractions of a temperate climate in August, with sufficient accommodations for the members who would probably be present. He urged the selection of Old Point Comfort, in Virginia, as the best place.

Dr. Brophy presented Mackinac, and said that it offered advantages which no other place could urge.

Dr. Cushing said that his correspondence had taught him that the southern members generally understood that there was an implied pledge that the Association should meet in 1888 at some point within the Southern States.

Dr. Barrett urged Old Point Comfort, and said that he believed a meeting at the South would prove of lasting benefit to dentistry, by bringing into more harmonious relations the members of the profession from the extremes of our country.

Old Point Comfort was chosen on the first ballot. The Association then proceeded to elect officers for the ensuing year, with the following result :

*President*—Frank Abbott, of New York.

*First Vice-President*—C. R. Butler, of Cleveland, Ohio.

*Second Vice-President*—T. S. Waters, of Baltimore, Md.

*Recording Secretary*—G. H. Cushing, of Chicago, Ill.

*Corresponding Secretary*—F. A. Levy, of Orange, N. J.

*Members of the Executive Committee*—A. W. Harlan, of Chicago; L. D. Shepard, of Boston; A. O. Hunt, of Iowa City.

A letter from Dr. J. Hall Moore, Chairman of the Executive Committee of the Southern Association was read, reciting the reasons why it was impossible for that society to meet with the Association in 1887, and expressing the desire that a union meeting should be arranged for 1888.

Dr. Barrett offered the following resolution, which was adopted :

*Resolved*—That the American Dental Association, recognizing the desirability of more intimate relations between the leading and representative professional organizations of American dentists, most cordially invites the Southern Dental Association to hold a union meeting with this Association next year. In furtherance of this end this Association has fixed upon a point for its next meeting which it believes will be convenient for both, and it desires that the committees of the two societies should agree upon a time that will be mutually satisfactory, and that this action will result in the largest and most profitable meeting of American dentists yet held.

Dr. Barrett then moved that the officers-elect of this Association be appointed delegates to the coming meeting of the Southern Association, with power to make all necessary arrangements. The motion was carried.

The consideration of the report from Section VI was then declared in order, when Dr. C. N. Peirce read a paper upon "The Evolution of the Human Dentition: A Comparison of the Development of the Teeth of Mammals."

In considering mammalian teeth, the essayist said it is necessary to have some knowledge of mammalian ancestry. The general structure of the earlier forms of life were compared and their analogy shown. In those mammals which have but one set of teeth, accepting the theory that the prototype is two dentitions, which of the two has been lost? Is it the deciduous or the permanent ones? The rudimentary character of the deciduous set would seem to indicate that they are the ones. The germs of the first permanent molars, arising *de novo,* are analogous in origin to the deciduous teeth. Children's teeth that are early erupted are not apt to possess strong recuperative or resistant powers.

*Dr. Hunt*—The writer of the paper seems to be in doubt whether "eruption" is the best term to use in speaking of the coming of the teeth. I think the term "emerge" will better express the condition, as "eruption" seems to imply a pathological rather than a physiological process.

*Dr. Brophy*—This affords an excellent opportunity for the Section on nomenclature to express its opinion, and I doubt not that it will present yet another term.

*Dr. Abbott*—My preference would be for the word "projection." If there were no continued growth at the end of the root, the teeth would not come forward. In cases of encysted tumors around the apex, there is no lengthening or procession of the tooth, and hence I believe that the appearance of the tooth above the gum is due to its projection by a force behind it.

Tomes claims that there is a special formation of osteoclasts which dissolve the ends of the roots when they are absorbed. I do not believe there is anything of the kind. He further says, that if absorption be due to pressure by the permanent teeth, that phenomena would be exhibited elsewhere. It is a fact that pressure on one side may produce absorption on the other. It is essentially an inflammatory process. As the permanent tooth is projected, it comes nearly in contact with the temporary root, and as a consequence of the pressure an inflammation ensues, which results in its absorption or melting down. The paper states that the absorption commences at the end of the root and proceeds directly onward. If the permanent tooth is projected at the side of the temporary one, absorption commences there.

*Dr. How*—Suggested the word "advance."

*Dr. Darby*—Neither of these words seems to convey the exact meaning. It would not be exactly proper to say that a child had advanced, or projected its teeth.

*Dr. Guilford*—Tomes declares the typal form of tooth to be a cone. Others pronounce it a wedge. How shall we account for the break between the different forms of teeth—the centrals, and the cuspids, and the bicuspids. Tomes believes that there was originally a tooth between the cuspid and the bicuspids. It has been asserted that the first permanent molar was primarily a persistent deciduous tooth.

*Dr. Peirce*—Dr. Black asserts that there is no perceptible difference in the absorption of the temporary and permanent teeth, but that which goes on in a tooth that has lost its pulp is quite a different affair. Adjourned.

(TO BE CONTINUED.)

